# Acute Traumatic Brain Injury Does Not Exacerbate Amyotrophic Lateral Sclerosis in the *SOD1*^G93A^ Rat Model^1^,^2^,^3^

**DOI:** 10.1523/ENEURO.0059-14.2015

**Published:** 2015-07-03

**Authors:** Gretchen M. Thomsen, Jean-Philippe Vit, Alexander Lamb, Genevieve Gowing, Oksana Shelest, Mor Alkaslasi, Eric J. Ley, Clive N. Svendsen

**Affiliations:** 1 Board of Governors Regenerative Medicine Institute, Cedars-Sinai Medical Center, Los Angeles, CA 90048; 2 Department of Biomedical Sciences, Cedars-Sinai Medical Center, Los Angeles, CA 90048; 3 Biobehavioral Research Core, Cedars-Sinai Medical Center, Los Angeles, CA 90048; 4 Department of Surgery, Cedars-Sinai Medical Center, Los Angeles, CA 90048

**Keywords:** ALS, amyotrophic lateral sclerosis, neurodegeneration, SOD1, TBI, traumatic brain injury

## Abstract

Amyotrophic lateral sclerosis (ALS) is a fatal motor neuron disease in which upper and lower motor neurons degenerate, leading to muscle atrophy, paralysis, and death within 3 to 5 years of onset. While a small percentage of ALS cases are genetically linked, the majority are sporadic with unknown origin. Currently, etiological links are associated with disease onset without mechanistic understanding. Of all the putative risk factors, however, head trauma has emerged as a consistent candidate for initiating the molecular cascades of ALS. Here, we test the hypothesis that traumatic brain injury (TBI) in the *SOD1* ^G93A^ transgenic rat model of ALS leads to early disease onset and shortened lifespan. We demonstrate, however, that a one-time acute focal injury caused by controlled cortical impact does not affect disease onset or survival. Establishing the negligible involvement of a single acute focal brain injury in an ALS rat model increases the current understanding of the disease. Critically, untangling a single focal TBI from multiple mild injuries provides a rationale for scientists and physicians to increase focus on repeat injuries to hopefully pinpoint a contributing cause of ALS.

## Significance Statement

Here we show that a one-time focal traumatic brain injury does not affect the disease time-course or survival in the *SOD1* ^G93A^ rat model of amyotrophic lateral sclerosis (ALS). This is important, as head injury has emerged as a strong candidate for initiating the neurodegenerative processes in ALS patients. By showing a lack of effect of acute, moderate/severe focal traumatic brain injury in this genetically predisposed model, focus can now be made on other types of central nervous system injuries, including mild repeat traumatic brain injury or diffuse axonal injury to elucidate the involvement of trauma in the initiation of *SOD1* mutation-based ALS.

## Introduction

Amyotrophic lateral sclerosis (ALS) is the most common motor neuron disease with progressive degeneration of motor neurons in the cortex, brainstem, and spinal cord. ALS patients undergo paralysis, respiratory insufficiency, and ultimately death typically within 3 to 5 years of disease onset. Point mutations in various genes, for instance the Cu/Zn *superoxide dismutase 1* (*SOD1*) and the more recently described *C9orf72* genes (Rosen et al., 1993; DeJesus-Hernandez et al., 2011; Renton et al., 2011), lead to familial forms of ALS. However, in the majority of patients, it is a sporadic disease of unknown origin.

No successful treatments exist for this devastating disease, in part because the mechanisms underlying the initiation of ALS pathology and subsequent motor neuron death have yet to be elucidated. This is because the etiology of ALS likely involves a complex interaction among multiple risk factors (Ling et al., 2013). One risk factor may be central nervous system (CNS) injury, given the recognized association between neurodegenerative disease and activities that involve a higher risk for CNS trauma, including participation in professional full-contact sports and in military service (Chio et al., 2009; Barnes et al., 2014; Mielke et al., 2014; Savica, 2014). In fact, CNS injury is linked to an increased incidence of motor neuron degeneration (Lehman et al., 2012; Li et al., 2014) and, thus, injury has emerged as a candidate that initiates the molecular cascades that yield neuronal death in ALS (Abel, 2007; Seelen et al., 2014).

CNS injury in humans and ALS rodent models elicits microglial activation in regions such as the spinal cord and brainstem (Alexianu et al., 2001; Turner et al., 2004; Evans et al., 2013). In terms of triggering disease spread, however, early acute glial activation in the spinal cord does not appear to hasten ALS progression. This is highlighted by ALS clinical trials that involve minor damage during spinal cord cell injections with no obvious acceleration of motor degeneration (Mazzini et al., 2012; Riley et al., 2014). In addition, a more severe injury due to damage following a stab-wound trauma to the *SOD1* rat spinal cord also did not accelerate motor neuron degeneration (Suzuki et al., 2010).

These injury studies, and ALS research in general, largely focus on the spinal cord with substantially less focus on the brain even though the cortical upper motor neurons are also vulnerable. Indeed, the brain plays an important role in initiating motor circuitry breakdown in ALS, not by overt cell death, but perhaps by dysfunctional actions that elicit system failure (Thomsen et al., 2014). Therefore, while the effect of injury on the spinal cord has been addressed in both sporadic and *SOD1*-linked cases, it is now critical to assess the role of brain injury given the novel finding that cortical dysfunction may be a key to motor circuitry breakdown in ALS.

Here we addressed the connection between an acute traumatic brain injury (TBI) and *SOD1* mutation-based ALS by using a transgenic rat model harboring a human *SOD1* gene mutation (*hSOD1* ^G93A^) that results in an ALS-like phenotype. Though correlative evidence exists to associate TBI and ALS, it remains unknown whether an effect on ALS may be linked to repeat injury or whether a single TBI is sufficient. As such, we tested whether a moderate or severe focal TBI administered one time to the *SOD1* ^G93A^ rat would lead to earlier onset of ALS, compromised motor function, and shorter lifespan in a genetically predisposed model. We found that a one-time focal trauma did not alter the time course of disease or death within this ALS rat model.

## Materials and Methods

### Animals

Sprague-Dawley wild-type (WT) and *SOD1* ^G93A^ (*SOD1*, herein) transgenic rats were housed under National Institutes of Health guidelines and all experiments were conducted in accordance with the Cedars-Sinai Institutional Animal Care and Use Committee (IACUC protocol 4260) and the Guide for the Care and Use of Laboratory Animals. This colony of transgenic rats provides later onset than the original model published by Howland and colleagues (Howland et al., 2002), with endpoint occurring at 180 ± 15.2 d. Reminiscent of human pathology, disease onset in hindlimbs and/or forelimbs is unpredictable and overt paresis progresses to complete paralysis. As it has been previously shown that male and female *SOD1* rats do not exhibit anatomical differences over time or show differences in disease onset or survival (Thomsen et al., 2014), both male and female rats were used in these studies (groups: 120 d, moderate: *n* = 4 WT TBI, *n* = 6 *SOD1* TBI, *n* = 6 *SOD1* sham; 90 d, severe: *n* = 4 WT sham, *n* = 6 WT TBI, *n* = 7 *SOD1* TBI, n = 8 *SOD1* sham).

### Controlled cortical impact injury

At the approximate age of 90 or 120 d, male and female presymptomatic rats were anesthetized with isofluorane and positioned within a rat stereotaxic frame. Following a left longitudinal scalp incision, a 6-mm-diameter craniotomy was made centered at bregma and 2.5 mm lateral to the midline. For rats at 120 d, “moderate” cortical injury was performed with a flat, 3-mm-diameter metal tip attached to the controlled cortical impact (CCI) device, at a velocity of 6 m/s, to a depth of 1.5 mm below the dura with a dwell time of 0.2 s. For rats at 90 d, “severe” cortical injury was performed at the same settings using a 4-mm-diameter tip and a depth of 2.5 mm.

### Tissue collection

Animals were euthanized by a ketamine/xylazine mixture administration followed by transcardial perfusion with 0.9% saline followed by 4% paraformaldehyde (PFA). Brain tissue was collected, postfixed in PFA overnight, and stored in 30% sucrose. Brains were sectioned at 35 μm using a microtome and collected as free-floating sections for histology.

### Contusion volume analysis

Brains were stained for cresyl violet and digital photographs of mounted sections were assessed for contusion volume using ImageJ software. Briefly, regions of interest were drawn around the entire contralateral and ipsilateral cortices and the percentage tissue loss was calculated by comparing the area of ipsilateral versus contralateral. Six brain sections per rat, spaced 240 μm apart were used for analysis.

### Motor behavior assessment

A blinded observer quantified several aspects of motor behavior, as defined below. Baseline behavioral testing was performed the week prior to injury and consisted of recording two sessions each of rotarod, grip strength (forelimb and hindlimb), and Basso, Beattie, and Bresnahan (BBB) analysis. Postinjury behavioral testing occurred on days 1, 3, and 7 after CCI and then continued weekly thereafter until *SOD1* rats began showing signs of disease, at which point BBB scoring (see below) increased to twice per week. *SOD1* rats along with age-matched WT controls were killed at disease endpoint (defined below).

*Rotarod.* To test balance and motility, rats were placed on a slowly rotating rod (3 inches in diameter; www.sandiegoinstruments.com) for 210 s per trial. The speed was set to start at 3 rpm and was constant for the first 30 s, then accelerated progressively for 3 min to reach the speed of 30 rpm. On each session day, the rats were given three trials separated by 30 min and the times spent on the rod were averaged for analysis. Rotarod testing was not performed for the experiment involving TBI at postnatal day (P) 120 in order to avoid unnecessary stress on the rats.

*Grip strength.* Each rat was allowed to grip with either forelimbs or hindlimbs a grid bar attached to a Chatillon digital force gauge (www.sandiegoinstruments.com) and was then gently pulled back until the bar was released. Three measurements of the peak force in grams for both forelimbs and hindlimbs were averaged for analysis.

*BBB locomotor rating scale*. The BBB locomotor rating scale (Basso et al., 1995) is used to assess an animal’s ability to walk around its environment and can quantify the degree of limb paralysis in *SOD1* ^G93A^ rats and mice. The 21-point BBB scoring is an open-field locomotor test of limb function, with a 21 score indicating coordinated limb movement, consistent toe clearance, and parallel paw placement, and a 0 score indicating no observable limb movement. BBB locomotor ratings provide an indication of when paralysis starts in any limb and the degree of progression continuing until the animal's endpoint. BBB scores and body weights were assessed once or twice weekly by an observer blinded for genotype and treatment, starting the week prior to cortical injury (which occurred at P90 or P120), and continuing until disease endpoint. Disease onset was classified as when an animal displayed a BBB score of 15 or lower. Endpoint was classified as when a rat was no longer able to “right” itself within 25 s of being placed on its side. At endpoint the animal will typically have lost 30% of its body weight and have a BBB score ≤5 in ≥1 limb.

### Statistical analysis

Statistical analyses were performed using Graph Pad Prizm software. Student’s *t*-tests and two-way ANOVA using Bonferroni’s post-hoc analyses were performed to determine SEM with a 95% confidence level. Kaplan–Meier survival curves were analyzed by the log-rank test and comparisons of median disease durations and survival times were analyzed by the Wilcoxon signed-rank test.

## Results

### A single moderate TBI administered at 120 d does not alter disease progression or lifespan

**Figure 1:**
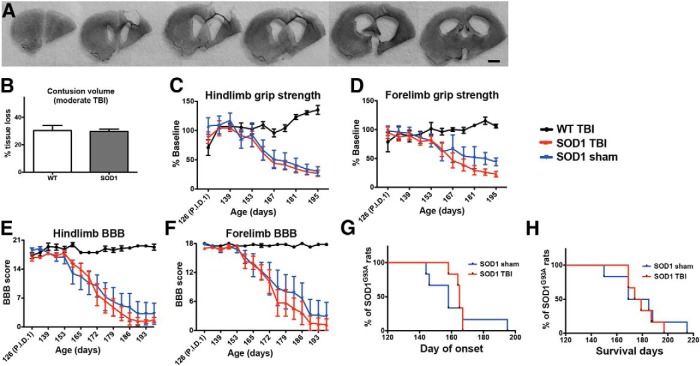
***A***, Coronal brain sections (420 µm apart, notched on the ipsilateral underside prior to sectioning) stained with cresyl violet from a rat that was administered a moderate CCI at P120. ***B***, Quantitative analysis of the cortical lesion site after TBI revealed that this moderate CCI injury resulted in an ∼30% loss of tissue in both WT and *SOD1* rats [30.5 ± 3.7% (SEM) in WT and 30.4 ± 1.1% in *SOD1* rats, ipsilateral tissue loss, relative to the contralateral cortex]. ***C–F***, This injury did not result in overt functional deficits in either WT or *SOD1* rats, relative to their sham counterparts, during the early time points after injury (time points P126–P153) as assessed by (***C***) hindlimb and (***D***) forelimb grip strength, as well as (***E***) hindlimb and (***F***) forelimb BBB scores. ***C–H***, Injured *SOD1* rats showed no differences in strength or motor function relative to *SOD1* sham rats at later time points (***C–F***) and there was no effect of TBI on (***G***) disease onset or (***H***) survival, supporting the idea that a one-time acute moderate TBI in rats close to disease onset does not predispose earlier onset or death. P.I.D. 1: post-injury day 1; Scale bar, 2 mm.

An association between head injury and increased incidence of neurodegeneration has been suggested (Mannix et al., 2013; Loane et al., 2014; Mielke et al., 2014). To test whether this is true for a population genetically predisposed to ALS, we assessed the effects of a single moderate TBI on disease onset, motor function, and lifespan in the *SOD1* rat model of ALS. CCI is an established method to induce a focal lesion in the cortex of a rodent model of TBI (Dixon et al., 1991, Goodman et al., 1994, Xiong et al., 2013). Baseline testing for strength and motor function the week prior to injury showed no differences between groups (data not shown). A moderate CCI administered unilaterally at P120 resulted in a cortical lesion with significant tissue loss (Fig. 1*A*). Cresyl violet staining showed that *SOD1* and WT had a comparable CCI, which is highlighted by the quantification of percent cortical tissue volume loss in Figure 1*B* (*p* = 0.906; Table 1, a). While the core of this injury was centered over the motor cortex at bregma, tissue damage was extensive and spanned from the frontal cortex, on average 3 mm anterior to bregma to 3 mm posterior to bregma (Fig. 1*A*). Although there was a significant, 30% loss of tissue (ipsilateral vs contralateral) in rats receiving a TBI, there were no observable functional deficits, and motor function as assessed by hindlimb/forelimb grip strength and hindlimb/forelimb BBB scores was similar among *SOD1* sham, *SOD1* TBI, and WT TBI groups in the early time period following injury (Fig. 1*C*–*F*, time points P126–P153; *p* = 0.689, 0.739, 0.270, 0.730, respectively; Table 1, b–e).

**Table 1:** Summary of statistics from figures

	Dataset	Data structure	Type of test	*p* value
a	Fig. 1B: lesion size column graph (WT vs TBI)	Normal distribution	*t*-test	0.906
b	Fig. 1C: hindlimb grip-strength graph; early postinjury time points	Normal Distribution	Two-way ANOVA: interaction	0.689
c	Fig. 1D: forelimb grip-strength graph; early postinjury time points	Normal distribution	Two-way ANOVA: interaction	0.739
d	Fig. 1E**:** hindlimb BBB graph; early postinjury time points	Normal distribution	Two-way ANOVA: interaction	0.27
e	Fig. 1F**:** forelimb BBB graph; early postinjury time points	Normal distribution	Two-way ANOVA: interaction	0.73
f	Fig. 1G: onset Kaplan–Meier curves	Normal distribution	Log-rank test	0.774
g	Fig. 1H: survival Kaplan–Meier curves	Normal distribution	Log-rank test	0.74
h	Fig. 1C: hindlimb grip-strength graph; all time points	Normal distribution	Two-way ANOVA: interaction	0.991
i	Fig. 1D: forelimb grip-strength graph; all time points	Normal distribution	Two-way ANOVA: interaction	0.955
j	Fig. 1E: hindlimb BBB graph; all time points	Normal distribution	Two-way ANOVA: interaction	0.986
k	Fig. 1F: forelimb BBB graph; all time points	Normal distribution	Two-way ANOVA: interaction	0.999
l	Fig. 2C: rotarod graph; early postinjury time points (WT: TBI vs sham)	Normal distribution	Two-way ANOVA: treatment	0.0006
m	Fig. 2C: rotarod graph; early postinjury time points (*SOD1*: TBI vs sham)	Normal distribution	Two-way ANOVA: treatment	<0.00001
n	Fig. 2D: hindlimb grip-strength graph; all time points (*SOD1*: TBI vs sham)	Normal distribution	Two-way ANOVA: treatment	<0.00001
o	Fig. 2E: forelimb grip-strength graph; all time points (*SOD1*: TBI vs sham)	Normal distribution	Two-way ANOVA: treatment	0.236
p	Fig. 2F: onset Kaplan–Meier curves	Normal distribution	Log-rank test	0.716
q	Fig. 2G**:** survival Kaplan–Meier curves	Normal distribution	Log-rank test	0.157
r	Fig. 2C**:** rotarod graph; all time points	Normal distribution	Two-way ANOVA: interaction	0.829
s	Fig. 2H: hindlimb BBB graph; all time points	Normal distribution	Two-way ANOVA: interaction	0.999
t	Fig. 2I: forelimb BBB graph; all time points	Normal distribution	Two-way ANOVA: interaction	0.97

Postinjury behavioral testing began on days 1, 3, and 7 after CCI and then continued weekly thereafter. While this moderate TBI did not produce an initial effect on motor function, we hypothesized that TBI would still affect disease onset, progression, and lifespan in the *SOD1* rat compared to *SOD1* sham rats. Assessment at later time points following injury, however, showed that the *SOD1* TBI rats had no difference in disease onset (*p* = 0.774; Table 1, f; Fig. 1*G*) or survival (*p* = 0.740; Table 1, g; Fig. 1*H*) relative to *SOD1* sham rats. In addition, injury did not cause a significant premature decrease of hindlimb (*p* = 0.991; Table 1, h; Fig. 1*C*) or forelimb (*p* = 0.955; Table 1, i; Fig. 1*D*) grip strength, and the BBB score, which is more closely related to the progression of ALS-associated paralysis, demonstrated no effects of TBI on either hindlimb (*p* = 0.986; Table 1, j; Fig. 1*E***)** or forelimb (*p* = 0.999; Table 1, k; Fig. 1*F*) function. As the injury was unilateral in the cortex, separate BBB analysis of right and left forelimb and hindlimb motor function was performed. The ipsilateral/contralateral ratios for disease onset and progression remained close to 1 during the course of disease in injured versus sham rats (data not shown), highlighting that the limbs associated with the injured brain region were not affected differently than the limbs associated with the uninjured brain region. Together, these results suggest that a single moderate TBI in the P120 *SOD1* rat does not compromise disease onset, motor function, or lifespan.

### A single severe TBI administered at 90 d does not alter disease progression or lifespan

**Figure 2:**
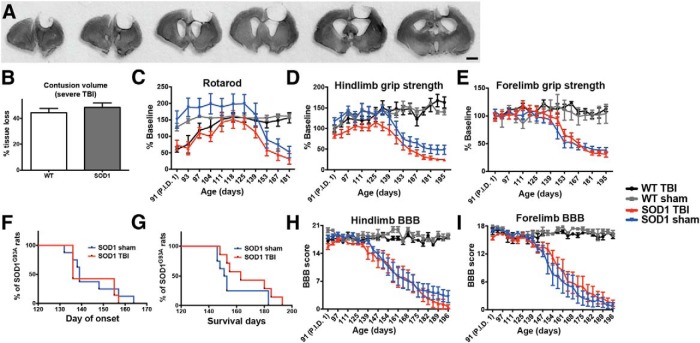
***A***, Coronal brain sections (420 µm apart, notched on the contralateral underside prior to sectioning) stained with cresyl violet from a rat administered a severe CCI at P90. ***B***, Quantitative analysis of the cortical lesion site after TBI revealed that this severe CCI injury resulted in an ∼45% loss of tissue in both WT and *SOD1* rats [44.3 ± 3.3% (SEM) in WT and 48.4 ± 3.5% in *SOD1*, ipsilateral tissue volume loss, relative to the contralateral cortex]. ***C***, This severe CCI injury resulted in significant early postinjury deficits (graph time points P91–P97) in both WT and *SOD1* injured rats, relative to their sham counterparts in rotarod performance. ***D***, ***E***, Relative to *SOD1* sham rats, injured *SOD1* rats showed a significant overall decrease due to injury in (***D***) hindlimb, but not (***E***) forelimb, grip strength. ***F–I***, However, measures of disease such as (***F***) onset, (***G***) survival, and (***H***) hindlimb and (***I***) forelimb BBB scores remained unchanged relative to *SOD1* sham controls, indicating that a one-time severe acute TBI at an early presymptomatic time point does not affect the disease onset or death in these rats. P.I.D. 1: post-injury day 1; Scale bar, 2 mm.

It is possible that older ALS rats receiving a moderate TBI were unaffected because this TBI model was not sufficiently severe and/or because the older age did not provide sufficient time for TBI-induced cellular changes to accumulate and thereby alter disease development. As such, we next assessed disease onset, motor function, and lifespan after a more severe CCI administered at an earlier time point (P90), in order to allow the downstream effects of the insult to accumulate over time. This injury resulted in a more significant loss of tissue than in the previous experiment (Fig. 2*A*), with cresyl violet staining and quantification of lesion size in both WT and *SOD1* TBI rats showing a 45% loss of cortical tissue (ipsilateral, relative to the contralateral cortex; Fig. 2*B*). While the core of this injury was centered over the motor cortex at bregma, tissue damage was extensive and spanned from the frontal cortex, on average 4 mm anterior to bregma to 4 mm posterior to bregma (Fig. 2*A*). Baseline testing the week prior to injury revealed no differences in motor function among any of the groups (data not shown). For this experiment, the rotarod test was added as an additional measure of motor function. Postinjury behavioral testing began on days 1, 3, and 7 after CCI and then continued weekly thereafter. Using this test, significant functional deficits resulting from TBI were observed, as rotarod performance of WT and *SOD1* rats with severe TBI during the early time points after injury was significantly lower compared to their respective baseline performance and relative to their sham counterparts (Fig. 2*C*, time points P91–P101; *p* = 0.0006 WT sham vs WT TBI, *p* < 0.00001 *SOD1* sham vs *SOD1* TBI; Table 1, l, m). Additionally, unlike previous behavioral results following only a moderate injury, *SOD1* rats with severe TBI were significantly weaker throughout the study in their hindlimbs (treatment effect *p* < 0.0001; Table 1, n; Fig. 2D) but not forelimbs (*p* = 0.236; Table 1, o; Fig. 2*E*), relative to *SOD1* sham controls. While these animals showed a clear reduction in overall hindlimb strength following a severe CCI, these behavioral changes did not translate into an earlier disease onset (*p* = 0.716; Table 1, p; Fig. 2*F*) or shortened lifespan (*p* = 0.157; Table 1, q; Fig. 2*G*) in *SOD1* rats with TBI relative to *SOD1* sham rats. Furthermore, *SOD1* rats receiving a severe TBI showed no premature decline in motor function compared with *SOD1* sham rats, as demonstrated by the rotarod performance (interaction of treatment × age *p* = 0.829; Table 1, r; Fig. 2*C*) and the BBB score for hindlimb (*p* = 0.999; Table 1, s; Fig. 2*H*) or forelimb function (*p* = 0.970; Table 1, t; Fig. 2*I*). Similar to the moderate TBI, as the injury was unilateral in the cortex, separate BBB analysis of right and left forelimb and hindlimb motor function was performed. The ipsilateral/contralateral ratios for disease onset and progression remained close to 1 during the course of disease in injured versus sham rats (data not shown), highlighting that the limbs associated with the injured brain region were not affected differently than the limbs associated with the uninjured brain region. Collectively, these results establish that even a severe TBI, administered to young *SOD1* rats to allow the effects of the insult to accumulate over time, did not alter ALS disease manifestation.

## Discussion

Clear, causative evidence linking traumatic events to incidences of ALS has not been established and our findings do not support an obvious involvement of acute focal brain trauma in triggering the onset or worsening of the disease in a genetically susceptible ALS population. While there is an established association between neurodegenerative disease and participation in professional sports, including boxing, football, and soccer, as well as military service, the common factors that cause this remain to be demonstrated (Chio et al., 2009; Barnes et al., 2014; Mielke et al., 2014; Savica, 2014). Though these activities are high risk for trauma to the CNS, which could be the common factor that triggers neurodegenerative pathology, other stressors might also be the cause. For instance, physical stress might be related, given the higher incidence of ALS in soccer players with lengthy careers and with midfield positions that require excessive running, as well as in tri-athletes (Beghi et al., 2010; Gotkine et al., 2014). This, however, remains controversial (Veldink et al., 2005; Pupillo et al., 2014).

While injury has emerged as a candidate for initiating the molecular cascades that yield neuronal death in ALS (Abel, 2007; Seelen et al., 2014), CCI did not affect disease onset in the *SOD1* rat model. While extrapolating from the rodent to the human condition requires caution, given that these rats are genetically predisposed to ALS using the human mutant *SOD1* gene, sufficient common underlying mechanisms likely make these rats a reliable representation for the lack of effect on disease progression following a single traumatic insult in the genetically susceptible population. An additional caveat comes from the fact that ALS is primarily a sporadic disease. It is possible that a one-time trauma elicits a different environmental interaction among sporadic ALS patients and it should be considered that these results are only directly related to this *SOD1* mutation-based ALS model. Therefore, additional, undefined environmental factors might be needed in conjunction with the acute brain injury in order to trigger or enhance disease spread. Finally, while the well-characterized *SOD1* rat is a widely accepted and used model of ALS (Howland et al., 2002), it is important to consider that other genetic mutations in addition to this *SOD1* ^G93A^ lead to familial onset. Therefore, while acute injury in this *SOD1* model did not affect disease manifestation, there could be an effect with different transgenic models of ALS.

The contribution of each major component of the motor neuron pathway (brain, spinal cord, muscle) to the origin of ALS remains unclear. Previous studies assessing the spinal cord have shown that subtle damage in ALS patients following needle injections (Mazzini et al., 2012; Riley et al., 2014) and even severe damage resulting from stab-wound trauma in the *SOD1* rat (Suzuki et al., 2010) did not accelerate motor neuron degeneration. We now demonstrate that acute damage to the brain does not accelerate disease onset, alter behavior, or shorten lifespan in this ALS rat model. The lack of effect following brain damage was surprising given the recent finding that the brain plays an important role in initiating motor circuitry breakdown in this same model (Thomsen et al., 2014). However, it may be that the brain is involved in ALS, not by overt cell death or cell loss (as induced with this CCI model), but rather by means of dysfunctional actions that then elicit system failure. Indeed, *SOD1* rats at P120 are behaviorally presymptomatic and do not show overt loss of corticospinal motor neurons but, critically, they already have significant loss of spinal motor neurons (Thomsen et al., 2014). This loss of motor neurons is likely the result of cellular stressors or toxic compounds that accumulate and possibly even spread in a prion-like fashion over time (Grad and Cashman, 2014; Polymenidou and Cleveland, 2011). This fits with a recent report showing a clear link between aging and the development of ALS (Das and Svendsen, 2015). Given these recent datasets, a late TBI may not provide time for deleterious effects of acute brain injury to accumulate and trigger disease manifestation. However, a more severe injury at early time points did not affect disease either and, therefore, if subtle cortical dysfunction is key to motor circuitry breakdown in ALS, a milder insult might trigger a system breakdown that is missed with the model of TBI used in this study, which is focal, severe, and causes a significant loss of brain tissue. This may not accurately represent athletes and veterans who may undergo milder and perhaps more repetitive instances of physical stress and/or trauma and therefore injury models that might better represent these conditions, such as diffuse axonal injury, or mild insults that are either repetitive or global (Young, 2002; Choo et al., 2009; Jin et al., 2014; Mierzwa et al., 2014), should still be assessed (Prins et al., 2010; Mannix et al., 2013; Angoa-Perez et al., 2014).

Correlative evidence has suggested that TBI is linked with ALS, but it is unknown whether a single injury is sufficient to hasten disease pathology. We show here that a one-time focal cortical trauma did not alter *SOD1* rat disease onset, progression, or lifespan. Given the importance of cortical dysfunction in initiating ALS (Thomsen et al., 2014), it was vital to uncover this minimal involvement of a single acute brain injury. While the role of multiple, milder brain injuries is still open for study, the new understanding that a single focal TBI does not exacerbate ALS, at least in a genetically predisposed population, narrows the search for this disease's elusive etiology.

## Synthesis

The decision was a result of the Reviewing Editor Jun Chen and the peer reviewers coming together and discussing their recommendations until a consensus was reached. A fact-based synthesis statement explaining their decision and outlining what is needed to prepare a revision is listed below. The following reviewer(s) agreed to reveal their identity: Dong Sun

Reviewer # 1 is satisfied with the revision "The negative findings correlating this specific ALS model with the specific TBI injury provide a reasonable check in the hypothesis that TBI exacerbates ALS in the SOD1 rat ALS model. The authors have amended the text sufficiently to avoid over interpretation given the ALS model that was used".

Reviewer# 2 thinks that as the manuscript reported negative data, careful interpretation of the data is needed. The revised manuscript addressed many of previous concerns; however, there are some additional minor concerns need to be addressed.

1) The TBI mode used in this study is a focal injury model. This needs to be clearly stated throughout particularly in the abstract in line 11-17 page 1, line 64/67 page 3.

2) In methods and results sections, it is not clear when the behavior tests were conducted following CCI, and how long the animals were left to survive following TBI.

3) Contusion volume assessment: not clear how many sections were assessed each brain; whether the same number of sections was included.

4) Fig. 1A and 2A only show brain sections taken from SOD CCI rats, suggest adding a panel of the same level pictures taken from the wild type animals with CCI for more direct comparison of brain sections.

Response to Reviewers

Thank you for reviewing our manuscript eN-NWR-0059-14R1 entitled "Acute traumatic brain injury does not exacerbate ALS in the SOD1G93A rat model" for publication in eNeuro. We appreciate the comments by the reviewers, and have made revisions to the manuscript taking into consideration all of their recommendations. Please see below for information regarding changes in the manuscript (found in BLUE text, original revisions found in RED text) based on the comments provided.

### Reviewer #1:

Reviewer # 1 is satisfied with the revision "The negative findings correlating this specific ALS model with the specific TBI injury provide a reasonable check in the hypothesis that TBI exacerbates ALS in the SOD1 rat ALS model. The authors have amended the text sufficiently to avoid over interpretation given the ALS model that was used".

### Reviewer #2:

Reviewer # 2 thinks that as the manuscript reported negative data, careful interpretation of the data is needed. The revised manuscript addressed many of previous concerns; however, there are some additional minor concerns need to be addressed.

1) The TBI mode used in this study is a focal injury model. This needs to be clearly stated throughout particularly in the abstract in line 11-17 page 1, line 64/67 page 3.

We have gone through the text and have clearly stated that this is a focal injury model by adding the word "focal" in the following places:

Abstract, lines 11-13

Significance Statement, line 17

Introduction, lines 64, 67

Discussion, lines 216, 271, 275

2) In methods and results sections, it is not clear when the behavior tests were conducted following CCI, and how long the animals were left to survive following TBI.

We have clarified this by adding the following statements to the Methods, lines 101-104 "Post-injury behavioral testing occurred on days 1, 3 and 7 after CCI and then continued weekly thereafter until SOD1 rats began showing signs of disease, at which point BBB scoring (see below) increased to twice per week. SOD1 rats were euthanized at disease endpoint (defined below) along with age-matched WT controls" and to the Results, lines 160-161 "Post-injury behavioral testing began on days 1, 3 and 7 after CCI and then continued weekly thereafter" and lines 191-192 "post-injury behavioral testing began on days 1, 3 and 7 after CCI and then continued weekly thereafter."

3) Contusion volume assessment: not clear how many sections were assessed each brain; whether the same number of sections was included.

We have clarified this by adding the following statement to the Methods, lines 96-97 "Six brain sections per rat, spaced 240um apart were used for analysis."

4) Fig. 1A and 2A only show brain sections taken from SOD CCI rats, suggest adding a panel of the same level pictures taken from the wild type animals with CCI for more direct comparison of brain sections.

We appreciate the Reviewer's comment about adding the CCI from wild type animals to Figure 1A and 2A, which currently shows the CCI from SOD1 rats. However, Figure 1B and 2B highlight that the CCIs between wild type and SOD1 animals are the same and we feel that including a qualitative panel of brain sections from wild type animals is redundant with the quantitative data already shown and, importantly, detracts from both Figures 1 and 2. To address the Reviewer's comment, we have, however, added the following sentences to the Results section (lines 149-151), "Cresyl violet staining showed that SOD1 and WT had a comparable CCI, which is highlighted by the quantification of percent cortical tissue volume loss in Fig. 1B," and (lines 185-186) "This injury resulted in a more significant loss of tissue than in the previous experiment (Fig. 2A), with cresyl violet staining and quantification of lesion size in both WT and SOD1 TBI rats showing a 45% loss of cortical tissue (ipsilateral, relative to the contralateral cortex, Fig. 2B)."
